# Relationship among Dexamethasone Suppression Test, personality disorders and stressful life events in clinical subtypes of major depression: An exploratory study

**DOI:** 10.1186/1475-2832-3-15

**Published:** 2004-12-14

**Authors:** KN Fountoulakis, A Iacovides, F Fotiou, M Karamouzis, A Demetriadou, G Kaprinis

**Affiliations:** 1 Lab of Psychophysiology, 3^rd ^Department of Psychiatry, Aristotle University of Thesssaloniki, Greece; 2 Lab of Clin Neurophysiology, 1^st ^Department of Neurology Aristotle University of Thesssaloniki, Greece; 3 Lab of Biochemistry, Aristotle University of Thesssaloniki, Greece

**Keywords:** Depression, stressful life events, stress, personality disorders, Dexamethasone suppression test.

## Abstract

**Background:**

The present study aimed to investigate the relationship between dexamethasone suppression test, personality disorder, stressful life events and depression.

**Material:**

Fifty patients (15 males and 35 females) aged 41.0 ± 11.4 years, suffering from Major Depression according to DSM-IV criteria entered the study.

**Method:**

Diagnosis was obtained with the aid of the SCAN v 2.0 and the IPDE. Psychometric assessment included the HDRS, HAS, the Newcastle Scale (version 1965 and 1971), the Diagnostic Melancholia Scale, the Personality Deviance Scale and the GAF scale. The 1 mg DST was used.

**Statistical Analysis:**

Included MANOVA, ANOVA with LSD post hoc test and chi-square test.

**Results:**

Sixteen (32%) patients were non-suppressors. Eight patients without Personality Disorder (PD) (23.5%), and 5 of those with PD of cluster B (50%) were non-suppressors. Atypical patients were the subtype with the highest rate of non-suppression (42.85%). No difference between suppressors and non-suppressors was detected in any of the scales.

**Discussion:**

The results of the current study suggest that pathological DST is not a core feature of major depression. They also suggest that there are more than one subtypes of depression, concerning the response to stress. It seems that the majority of depressed patients (50%) does not experience high levels of stress either in terms of self reported experience or neuroendocrine function. The rest of patients however, either experience high levels of stress, or manifest its somatic analogue (DST non-suppression) or have a very low threshold of stress tolerance, which makes them to behave in a hostile way.

## Background

Life events and environmental stressful factors may relate to the development of depression [1-4]. However, biological theories suggest that the cause of depression rely on a biochemical disturbance of the functioning of the central nervous system (CNS).

The Dexamethasone Suppression Test (DST) [5] is the most known and worldwide used biological marker, its results suggest that a disorder of the HPA axis is present in at least some depressed patients [6]. DST non-suppression is of unknown aetiology, and as a test is not specific to any disease. Rather it constitutes an endocrin expression of stress. Basically, DST is reported to assess norepinephrine function. Topographically, it assesses the function of the hypothalamus and indirectly of the structures, which project to it. However, it is also supposed to be the result of an increased serotonin (5-HT) or Ach activity, or of a disturbance of the feedback to the hippocampus [7] and the hypothalamus. A debate still holds, whether some forms of depression are characterized by hypercortisolaimia or early escape from HPA tests. Possibly, DST non-suppression and hypercortisolemia are two different things [8].

The present study aimed to investigate the relationship between dexamethasone suppression test, personality disorder (PD), stressful life events and clinical manifestations of major depression. The hypothesis to test was that subtypes of depression could be identified on the basis of the presence of personality disorder (which constitutes an abnormal interpretation and response to environmental stimuli), the presence of abnormal DST results and/or hypercortisolemia (which both constitute an idiosyncratic neuroendocrine response to stress) and the presence or not of stressful life events (which trigger the above behavioral and neuroendocrine responses).

The presence or not of Personality Disorder, and the response to the DST are both characteristics of the patient. Life events reflect the impact of the environment on the patient. So, life events provoke responses from the side of the patient, which are largely determined by Personality and DST response. Thus, four groups of patients can be identified and studied, according to the combination of the co-existence of DST non-suppression and personality disorder.

## Material

Fifty (50) major depressive patients (15 males and 35 females) aged 41.0 ± 11.4 (range 21–60) years [9,10], took part in the study. All provided written informed consent. Fourteen of them fulfilled criteria for atypical features, 16 for melancholic features (according to DSM-IV) and 32 for somatic syndrome (according to ICD-10). Nine patients did not fulfil criteria for any specific syndrome according either classification system.

Patients were in- or outpatients of the 3^rd ^department of psychiatry, Aristotle University of Thessaloniki, Greece. They constituted consecutive cases that fulfilled the inclusion criteria and no systemic bias exists.

The SCAN v 2.0 [11] was used for the diagnosis of depression and its subtypes and the IPDE [12-14] was used for the diagnosis of personality disorders.

Seventeen patients (34%) suffered from a personality disorder (PD). Ten of them (20%) had a cluster B PD. Concerning depressive subtypes, 5 (out of 16) melancholics (26.32%), 7 (out of 14) atypicals (50%), 9 (out of 32) patients with somatic syndrome (28.13%), and 3 (out of 9) 'undifferentiated' patients (33.33%), fulfilled criteria for PD (note: patients with PD are not 5 + 7 + 9 + 3 = 24, but only 17 as mentioned above, because there is ovelapping between depressive syndromes). No patient suffered from a paranoid, schizotypal, antisocial, dissocial, narcissistic, and avoidant PD, although individual criteria were met. No criteria belonging to the schizotypal or antisocial PDs were met.

No patient fulfilled criteria for catatonic or psychotic features or for seasonal affective disorder. No patient fulfilled criteria for another DSM-IV axis-I disorder, excepting generalized anxiety disorder (N = 10) and panic disorder (N = 7). Another 5 patients had both generalized anxiety disorder and panic disorder (totally 22 patients that is 44% had some anxiety disorder).

The present study did not include a normal controls group, since the aim of the study was to compare depressive subtypes between each other.

### Method

**Laboratory Testing **included blood and biochemical testing, test for pregnancy, T3, T4, TSH, B_12 _and folic acid.

**The Psychometric Assessment **included the Hamilton Depression Rating Scale (HDRS), the Hamilton Anxiety Scale (HAS), the 1965 and 1971 Newcastle Depression Diagnostic Scale (1965 and 1971-NDDS) and the Diagnostic Melancholia Scale (DMS) [15] and the General Assessment of Functioning Scale (GAF) [16]. An attempt was made to assess the direction of aggression of the depressed patients, with the use of the Personality Deviance Scale (PDS) [17]. This was done mainly because the direction of aggression is considered to be a core feature for the etiopathogenesis of depression according to psychodynamic theories, but also is related to personality traits.

The PDS consists from the following subscales:

a. Extrapunitive Scale (ES) which consists of 1. HT: Hostile Thoughts and 2. DO: Denigratory Attitudes Toward other People. All these scales and subscales are scored in such a way that high scores denote lack of the characteristic.

b. Intropunitive Scale (IS), which consists of 1. LSC: Lack of Self-Confidence and DEP: Overdependency on Others. All these scales and subscales are scored in such a way that high scores denote presence of the characteristic.

c. Dominance Scale (DS) which consists of 1. MIN: Domineering Social Attitude and 2. HA: Uninhibited Hostile Acts. The MIN is scored in such a way that high scores denotes presence of the characteristic, while HA has opposite properties.

### Data concerning personal and family history and stressful life events

a. age of onset b. presence of a recent suicide attempt c. history of such attempts d. The questionnaire of Holmes [18] was used to search for stressful life events during the last 6 months before the onset of the symptomatology.

The 1 mg **Dexamethasone Suppression Test (DST) **protocol demands the administration of 1 mg dexamethasone per os at 23.00 of the first day, and determination of cortisol serum levels simultaneously and the next day at 16.00 and 23.00. Cortisol levels expressed in μg/dl were measured with Luminance Immunoassay (intra-essay reliability: 4.9%; inter-essay: 7.5%). Non-suppression cut-off level: 5 μg/dl.

### Statistical Analysis

Multiple Analysis of Variance (MANOVA) was performed with DST (suppression vs. non suppression) and Personality Disorder (present vs. absent) as factors. The dependent variables list included: Age, Age of Onset, Number of previous episodes, Number of DSM-IV Criteria, Number of atypical features, Number of melancholic features, GAF, NDDS 1965, NDDS 1971, Endogenous axis of DMS, Reactive axis of DMS, Number of stressful life events, HDRS-17, HDRS-21, HDRS Depressive index, HDRS Anxiety index, HDRS Sleep index, HDRS non-specific index, HAS, HAS Somatic subscale, HAS Psychic subscale, PDS-Hostile Thoughts Scale, PDS-Denigratory Attitude Scale, PDS-Extrapunitive Scale, PDS-Low Self Confidence Scale, PDS-Overdependency by others Scale, PDS-Intropunitive Scale, PDS-Domineering Social Attitude Scale, PDS-Uninhibited Hostile Acts Scale and PDS-Dominance Scale.

Afterwards, Analysis of Variance (ANOVA) with Least Significance Difference (LSD) test as post-hoc test was performed.

Finally, Chi-square test was performed. PD and DST were independently placed in cross-tabulation with the presence or absence of Recent Suicide Attempt, History of Suicide Attempt, Generalized Anxiety or Panic Disorder, Melancholic Features, Atypical Features, Somatic Syndrome, 'Undifferentiated' symptomatology, Full and sustained remission, With Relapsing circumscribed episodes, Chronic Depression without full remission, Presence of Stressful life events, Family history of any mental disorder, Family history of depression in 1^st ^degree relatives, and Family history of depression in 2^nd ^degree relatives.

## Results

Women were twice as many as men (70% versus 30%), which is not uncommon [19] and reflects the higher prevalence of depression observed in women.

Sixteen out of 50 depressed patients (32%) were DST non-suppressors (NS).

Eight out of 17 (47.05%) depressed patients with PD were also NS.

When the patients with a coexistent personality disorder (PD) were excluded, then 8 out of 33 (24.24%) patients left, were NS.

When only cluster b PDs were excluded, the respected percentage of NS climbs to 27.5% (11 out of 40).

Fifty percent of Cluster b PD patients were NS (5 S and 5 NS).

Six out of 14 (42.85%) atypical patients were NS, and this percentage makes this subtype the one with the highest NS percentage.

No one of Chi-square tests revealed any significant findings (at p > 0.01).

MANOVA results were significant both for Personality Disorder (p < 0.001) and for DST (P < 0.001) (table 1).

**Table 1:** 2-way MANOVA results. Both Personality disorders and DST results and their interaction produce significant results.

	**Wilks' Lambda**	**Rao's R**	**df 1**	**df 2**	**p-level**
**Factors:** **1-Personality Disorder (present vs. absent) and** **2-DST results (suppressors vs. non-suppressors)**					
1	0.02	18.26	30	12	***0.000***
2	0.02	20.99	30	12	***0.000***
12	0.01	28.42	30	12	***0.000***

**Figure 1:**
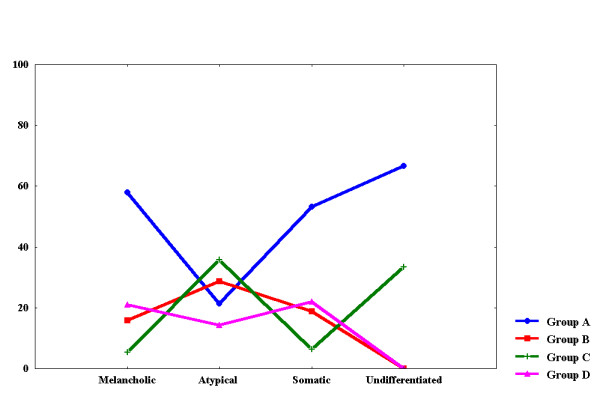
Histogram of the Distribution of Frequencies of Depressive Subtypes in the Four Groups

**Figure 2:**
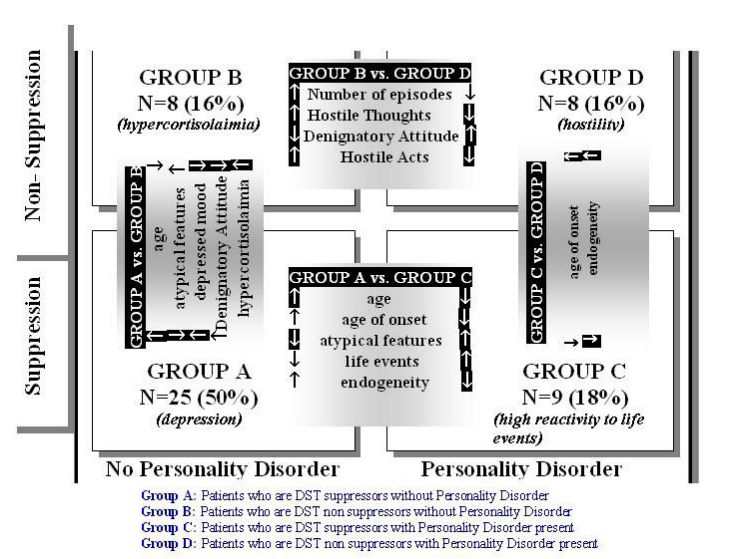
Characteristics of the four groups (white arrows in dark background indicate that the characteristic takes its largest or lower value in the respective group in comparison to all 4.

ANOVA testing, separately for each dependent variable, revealed significant findings concerning the number of episodes, and HT, DO and HA subscales of the PDS. When PD was used as the sole factor variable, significant findings were found concerning the endogenous axis of DMS and the HDRS depressive index. The interaction of PD and DST produced significant findings concerning age, age of onset, number of atypical features, number of stressful life events, and the DO subscale of the PDS (table 2). Post-hoc comparisons for DST showed that NS were more endogenous (1971-NDDS and DMS endogenous axis) but with lower HDRS depressive index (p < 0.05). Post-hoc comparisons for PD characteristics showed that patients without PD had more previous episodes and less hostile thoughts (HT) and less uninhibited hostile acts (HA) (p < 0.05). The post-hoc results for the groups defined by the interaction of PD with DST are shown in table 3. A graphical representation of these results is shown in figures 1 and 2.

**Table 2:** ANOVA results for each dependent variable separately (only significant results are shown.

	**df Effect**	**MS Effect**	**df Error**	**MS Error**	**F**	**p-level**
**Factors**: **1-Personality Disorder (present vs. absent) and** **2-DST results (suppressors vs. non-suppressors)**						

**Dependent variable: age**						
1	1	93.29	46.00	103.25	0.90	0.347
2	1	80.23	46.00	103.25	0.78	0.383
12	1	935.13	46.00	103.25	9.06	***0.004***
**Dependent variable: endogenous axis of DMS**						
1	1	9.08	46.00	8.26	1.10	0.300
2	1	78.71	46.00	8.26	9.53	***0.003***
12	1	21.10	46.00	8.26	2.55	0.117
**Dependent variable: age of onset**						
1	1	71.51	46.00	117.92	0.61	0.440
2	1	82.59	46.00	117.92	0.70	0.407
12	1	750.95	46.00	117.92	6.37	***0.015***
**Dependent variable: number of episodes**						
1	1	17.46	46.00	2.11	8.28	***0.006***
2	1	0.48	46.00	2.11	0.23	0.637
12	1	0.31	46.00	2.11	0.15	0.703
**Dependent variable: number of atypical features**						
1	1	0.81	46.00	0.75	1.09	0.302
2	1	0.59	46.00	0.75	0.79	0.377
12	1	4.35	46.00	0.75	5.82	***0.020***
**Dependent variable: number of stressful life events**						
1	1	10.45	46.00	3.27	3.20	0.080
2	1	4.87	46.00	3.27	1.49	0.229
12	1	19.51	46.00	3.27	5.97	***0.018***
**Dependent variable: HDRS Depressive Index**						
1	1	1.47	46.00	7.04	0.21	0.650
2	1	44.23	46.00	7.04	6.29	***0.016***
12	1	4.01	46.00	7.04	0.57	0.454
**Dependent variable: PDS HT subscale**						
1	1	76.28	41.00	9.74	7.83	***0.008***
2	1	4.23	41.00	9.74	0.43	0.514
12	1	10.51	41.00	9.74	1.08	0.305
**Dependent variable: PDS DO subscale**						
1	1	44.95	41.00	10.11	4.44	***0.041***
2	1	10.27	41.00	10.11	1.02	0.319
12	1	40.50	41.00	10.11	4.01	***0.052***
**Dependent variable: PDS HA subscale**						
1	1	97.48	41.00	13.12	7.43	***0.009***
2	1	7.91	41.00	13.12	0.60	0.442
12	1	30.77	41.00	13.12	2.35	0.133

**Table 3:** Post-hoc comparison between the four diagnostic groups determined by DST results and the presence of personality disorder concerning the continuous variables (Least Significance Difference-LSD Test).

	**Group A**		**Group B**		**Group C**		**Group D**							
	**N = 25 (50%)**		**N = 8 (16%)**		**N = 9 (18%)**		**N = 8 (16%)**		**p**	**p**	**p**	**p**	**p**	**p**

	**Mean**	**SD**	**Mean**	**SD**	**Mean**	**SD**	**Mean**	**SD**	**A/B**	**A/C**	**A/D**	**B/C**	**B/D**	**C/D**

Age	44.90	9.55	34.00	10.89	33.78	8.96	40.57	11.63	***0.005***	***0.002***	0.168	0.964	0.241	0.173
Age of Onset	33.33	11.24	29.00	10.74	23.44	7.13	35.00	13.14	0.217	***0.009***	0.967	0.223	0.313	***0.028***
Number of Episodes	1.52	1.89	1.88	1.55	0.33	0.71	0.43	0.53	0.575	0.068	0.092	***0.017***	***0.021***	0.893
Number of atypical features	0.71	0.85	1.63	1.06	1.67	1.00	1.14	0.38	***0.019***	***0.010***	0.102	0.935	0.375	0.298
DMS Endogenous axis	4.33	2.29	5.88	1.89	2.11	2.52	6.57	4.28	0.217	***0.032***	0.155	***0.004***	0.754	***0.018***
Number of Life Events reported	2.05	0.97	2.50	2.39	4.22	2.77	2.14	1.77	0.260	***0.001***	0.529	0.193	0.720	0.082
HDRS depressed index	11.43	2.38	8.50	2.14	10.22	3.87	8.86	2.79	***0.005***	0.350	***0.014***	0.282	0.837	0.378
HT	19.24	2.36	19.63	2.56	17.44	3.88	15.71	4.50	0.703	0.129	***0.012***	0.197	***0.045***	0.422
DO	13.00	3.16	9.88	3.44	13.11	2.57	14.14	3.63	***0.028***	0.927	0.431	***0.043***	***0.036***	0.515
HA	18.86	3.61	19.75	3.28	17.44	4.90	14.71	1.25	0.548	0.385	***0.007***	0.279	***0.002***	0.175
DST baseline cortisol value (day 1, 23:00)	3.85	2.79	7.71	10.28	3.79	1.71	5.43	4.37	0.123	0.724	0.568	0.275	0.491	0.474
DST cortisol level at day 2, 16:00	1.40	1.13	6.81	7.91	1.34	0.98	4.84	5.32	***0.002***	0.973	***0.001***	0.057	0.584	***0.047***
DST cortisol level at day 2, 23:00	1.25	1.45	8.04	5.19	1.36	0.71	5.13	1.40	***0.000***	0.769	***0.000***	***0.002***	0.212	***0.000***

**Group A: **DST suppressors, no PD

**Group B: **DST non-suppressors, no PD

**Group C: **DST suppressors, with PD

**Group D**: DST non-suppressors, with PD

DST suppressors without PD were older, with more severe depressed mood and less atypical features (50% of patients, figure 2, group A).

DST non-suppressors without PD were hypercortisolemic, with less severe depressed mood and denigratory attitude towards others (16% of patients, figure 2, group B).

DST suppressors with PD were younger, with younger age of onset, more atypical features and less endogeneity and more stressful life events (18% of patients, figure 2, group C).

DST non-suppressors with PD had older age of onset, high endogeneity and high levels of expressed hostility (16% of patients, figure 2, group D).

## Discussion

The current study reports that personality disorders (PD) in depressed patients is 2.5–3 times higher in comparison to the general population. Half (47.05%) of these PD patients were also DST non-suppressors (NS). Atypical patients was the depressive subtype with the highest frequency of both personality psychopathology and DST NS.

Figure 2 represents a graphical image of the intercorrelations between personality disorder, DST results and clinical manifestations. It seems that there is a circular relationship between PD, DST, age at interview, age of onset, number of episodes, reactivity to environment, hostility and depressed mood.

DST results seem to be a severity marker rather than directly related to symptomatology. In patients without PD, DST NS (group B in figure 2) may relate to milder depressed mood, higher denigratory attitude and hostility, higher number of previous episodes and hypercortisolemia. In patients with PD, non suppression (group D in figure 2) was related to 'endogenous quality' of depression, and higher levels of hostility. These patients (group B) are highly hostile and perform uninhibited hostile acts, however simultaneously have lower denigratory attitude and hostile thoughts (possibly the hostility is impulsive) and older age of onset.

Half of depressed patients belonged to the A group (suppressors without PD), and were characterized by the absence of atypical features. One could say that they represent a more 'formal' group of depressed patients. The rest of patients were equally distributed in the three groups (B, C and D). Groups B and C may represent two distinct types of vulnerability to stress (hypercortisolemia, DST non suppression and PD), while group D seems to represent a more severe form of depression, with an 'autonomous' hostility independent from the environment. This severe type could be considered to be the product of the accumulation of both vulnerabilities that characterize groups B and C, with the addition of a very low threshold for the tolerance of stress.

Nearly 4–10% of normal persons are reported to be DST-NS. The reason for this is unknown, however it has been suggested that it is due to an underlying mood disorder or family history of affective disorder. Another explanation suggests that DST reflects in fact the degree of psychological pressure or discomfort of the subject and not a specific vulnerability or characteristic of depression. It seems that non-suppression is gradually increasing along a continuum, which has mourning outpatients on the one pole (13% NS) and severe psychotic melancholic inpatients with psychotic features and suicidal ideation on the opposite one (64% NS) [20]. In this frame, the percentage of non-suppression reported in the current study (32%) is not in contrast with the international literature, since most of patients were out-patients and 16 of them (32%) were melancholics. An important finding is the 42.85% rate of non-suppression in atypical patients. This is reported for the first time in the international literature.

DST NS and hypercortisolemia may constitute two separate entities. For example, a patient may have baseline cortisol equal to 6 μg/dl, second cortisol value equal to 2.5 μg/dl and third cortisol value equal to 5.5 μg/dl and thus is classified as NS, but is not hypercorisolemic. On the contrary, a patient with baseline cortisol value equal to 10 μg/dl, second value equal to 4 μg/dl and third also equal to 4 μg/dl, is classified as NS, but is hypercorisolaimic.

Kirschbaum et al [21] reported that it is possible, some normal control subjects do not manifest the hypercorisolaimic response to stressful life events when these events are repeated (habituation). They also divided responses in high and low-cortisol responses. They related the first group with low self-confidence, increased depressed mood and higher number of symptoms, and the second group with lower extraversion. Joyce et al [22] suggested that the hypercortisolaimic response is related to a tendency for dependence and extravagance. These are generally in accord with the findings of the present study.

In contrast to what is widely accepted, NS is appeared to be closer to the atypical subtype. There are no direct reports in the international literature on this matter. However, the results of the study of Kocsis et al [23], in essence are in accord with the current study.

Rothschild et al [24] related DST NS with increased dopamine (DA) activity. Atypical patients, on the other hand, when compared with melancholics, reported more stressful life events, relatively higher levels of anxiety and shorter brain potentials [25]. While it is not possible to interpret what is the cause and what is the effect, it is interesting that there are papers in the international literature suggesting that conditions of internal conflict increase DA activity and lead to the appearance of displacement activities, which in turn serve the lowering of the level of arousal and stabilize the system [26]. Increased appetite, food intake and weight gain (atypical features) could be attributed to such a displacement activity. From the opposite point of view, the exhaustion of DA storage is reported to increase vulnerability to stress, because the already hyperfunctioning neurons (DST non-suppression) fail to respond properly [27]. According to Tazi et al [26], behavioral analogues of the defensive mechanism of displacement seem to suppress this procedure and in this way contribute to the better copying with stressful situations.

## Conclusion

Although the study sample of the current study is relatively small, the results suggest that there are more than one subtypes of depression, concerning the response to stress. The majority of depressed patients (50%) seems not to experience high levels of stress both in terms of self reported experience and neuroendocrine function. The rest of patients however, experience high levels of stress, either internally or have the somatic analogue of it (DST non-suppression) or have a very low threshold of stress tolerance, which makes them to behave in a hostile way.

## Competing interests

The authors declare that they have no competing interests.
